# Identification of Novel Prognostic Markers Associated With Laryngeal Squamous Cell Carcinoma Using Comprehensive Analysis

**DOI:** 10.3389/fonc.2021.779153

**Published:** 2022-01-11

**Authors:** Chao Huang, Jun He, Yi Dong, Li Huang, Yichao Chen, Anquan Peng, Hao Huang

**Affiliations:** ^1^ Department of Otolaryngology-Head and Neck Surgery, Second Xiangya Hospital Central South University, Changsha, China; ^2^ Department of Nephrology, Xiangya Hospital Central South University, Changsha, China; ^3^ Department of Cell Biology, School of Life Sciences, Central South University, Changsha, China; ^4^ Hunan Key Laboratory of Organ Fibrosis, Central South University, Changsha, China; ^5^ National Clinical Research Center for Geriatric Disorders, Xiangya Hospital Central South University, Changsha, China

**Keywords:** laryngeal squamous cell carcinoma, WGCNA, differentially expressed genes, prognostic gene, gene modules, single-cell analysis, TEDC2

## Abstract

**Background:**

Laryngeal squamous cell carcinoma (LSCC) is a leading malignant cancer of the head and neck. Patients with LSCC, in which the cancer has infiltrated and metastasized, have a poor prognosis. Therefore, there is an urgent need to identify more potential targets for drugs and biomarkers for early diagnosis.

**Methods:**

RNA sequence data from LSCC and patients’ clinical traits were obtained from the Gene Expression Omnibus (GEO) (GSE142083) and The Cancer Genome Atlas (TCGA) database. Differentially expressed gene (DEG) analysis and weighted gene co-expression network analysis (WGCNA) were performed to identify hub genes. Gene ontology (GO), Kyoto Encyclopedia of Genes and Genomes (KEGG) analysis, prognostic value analysis, receiver operating characteristic (ROC) curve analysis, gene mutation analysis, tumor-infiltrating immune cell abundance profile estimation, gene set variation analysis (GSVA), and gene set enrichment analysis (GSEA) were performed. Single-gene RNA sequencing data were obtained from the GSE150321 dataset. Cell proliferation and viability were confirmed by the CCK-8 assay and real-time PCR.

**Results:**

A total of 701 DEGs, including 329 upregulated and 372 downregulated genes, were screened in the GSE142083 dataset. Using WGCNA, three modules were identified to be closely related to LSCC. After intersecting the DEGs and performing univariate and multivariate Cox analyses, a novel prognostic model based on three genes (*SLC35C1*, *HOXB7*, and *TEDC2*) for LSCC was established. Interfering *TEDC2* expression inhibited tumor cell proliferation and migration.

**Conclusions:**

Our results show that *SLC35C1*, *HOXB7*, and *TEDC2* have the potential to become new therapeutic targets and prognostic biomarkers for LSCC.

## Introduction

Laryngeal squamous cell carcinoma (LSCC) is a common type of head and neck squamous cell carcinoma (HNSCC), accounting for approximately 20% of all cancer patients and 2.4% of new malignancies worldwide each year (1–3). Patients with LSCC, in which the cancer has infiltrated and metastasized, have a poor prognosis with a 5-year survival rate of approximately 60% (4). Despite recent advances in comprehensive surgical, chemotherapeutic, and radiotherapeutic treatment strategies, the global mortality rate associated with LSCC has not decreased (5). There are almost no typical symptoms in the early stages of LSCC, and mild symptoms are almost always ignored by patients (6). Therefore, the identification of abnormally expressed genes in LSCC and early intervention are important strategies for prolonging the survival time of patients with LSCC.

In recent years, with the development of gene chip technologies, methods of cancer diagnosis have become increasingly more efficient and considerably simpler, allowing researchers to improve cancer diagnoses in a relatively short period of time, aiding the identification of improved treatment measures (7–9). Additionally, owing to these advantages, research into specific molecular markers of cancer has become a topic of increased interest in recent years (10–13). Weighted gene co-expression network analysis (WGCNA) is a powerful approach for identifying gene co-expression modules, exploring the correlation between the modules and phenotypes and discovering hub genes that regulate critical biological processes (14–16). However, only few reports in the literature describing the hub genes and biomarkers in LSCC patients were identified by WGCNA (17–20).

In this study, a transcriptome dataset of LSCC and adjacent normal tissues from patients was obtained from the Gene Expression Omnibus (GEO) and used to identify the differences in expression profiles and the differentially expressed genes (DEGs) between the LSCC and control groups using WGCNA. A prognostic model of LSCC was established using The Cancer Genome Atlas Head and Neck Squamous Cell Carcinoma (TCGA-HNSC) dataset. Single-cell RNA-sequencing (scRNA-Seq) data from GEO were also used to verify the prognostic model and the expression profile of prognostic genes in different cell types. The present study aimed to improve our understanding of the pathogenesis of LSCC by identifying the underlying specific molecular mechanisms and to provide insights into novel therapeutic drug targets for the treatment of LSCC.

## Methods

### Data Collection and Single-Cell RNA-Sequencing Data Processing

**Figure 1:**
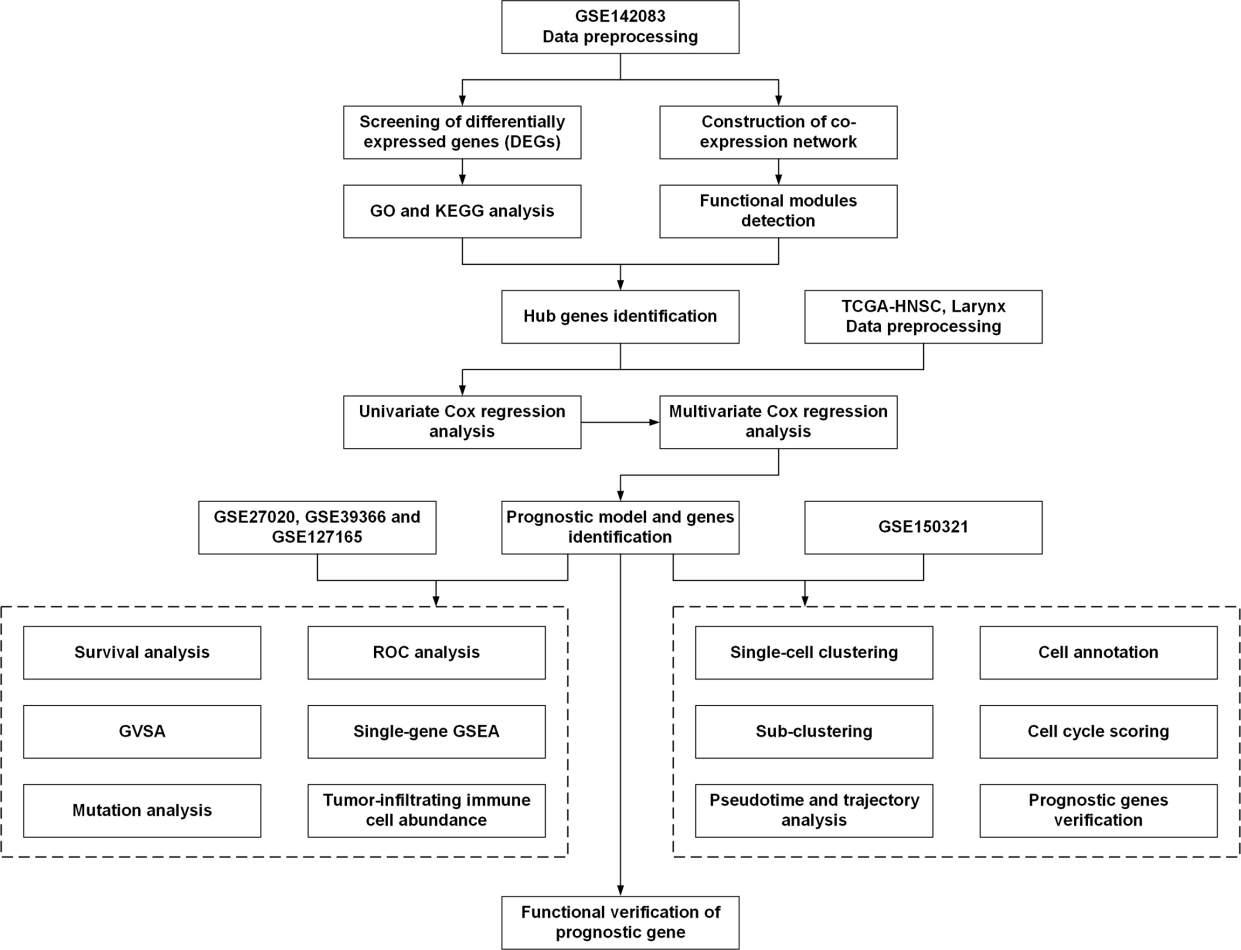
The workflow of the present study. DEGs, differentially expressed genes; GO, Gene Ontology; KEGG, Kyoto Encyclopedia of Genes and Genomes; TCGA, The Cancer Genome Atlas; ROC, receiver operating characteristic; GSEA, gene set enrichment analysis; GSVA, gene set variation analysis.

The workflow used in this study is shown in **Figure 1**. The search words “laryngeal carcinoma” OR “LSCC” OR “laryngeal cancer” AND “human” AND “Expression profiling by array” were applied for dataset retrieval. Datasets that included non-tumor samples and the total sample number of over 100 could be selected. Therefore, GSE142083 was selected to perform the following bioinformatics analysis. The gene expression matrix of 106 LSCC samples (53 LSCC and paired adjacent normal mucosa tissues) was downloaded from the GSE142083 dataset obtained from the GEO database (21). The GEO expression matrix was annotated with gene symbols using information from the GPL20301 Illumina HiSeq 4000 (Homo sapiens) platform file (https://www.ncbi.nlm.nih.gov/geo/query/acc.cgi?acc=GPL20301) as transcripts per million (TPM), and this information was log_2_ transformed in R (version 4.0.4) and RStudio (version 1.2.5033) when necessary. Principal component analysis (PCA) using the “ggfortify” package was performed, and the outliers were excluded (GSM4219698 and GSM4219730) (**Supplementary Figure 1**). The remaining data from the 52 LSCC and 52 paired normal tissue samples were included in the subsequent analysis. GSE27020 (22), GSE39366 (23), and GSE127165 (24) were also downloaded from GEO for validation.

The gene expression RNA-Seq data and the corresponding clinical data from the TCGA-HNSC dataset were downloaded from the University of California, Santa Cruz (UCSC) Xena (http://xena.ucsc.edu/).

scRNA-Seq data from the GSE150321 dataset were obtained from the GEO database (25). The “Seurat” R package (version 4.0.2) was used to process the data (26). The scRNA-Seq data of GSM4546857 and GSM4546858 from two LSCC patients were included and processed as described previously. Cells with less than 200 genes detected genes were excluded. Data were normalized by the method “LogNormalize” with a scale factor equal to 10,000. After determining the statistically significant principal components, cell clusters were annotated using the information from previous literatures. Next, we implemented the t-distributed stochastic neighbor embedding (t-SNE) algorithm to explore and visualize the cluster classification across cell samples. The trajectory analysis and pseudotime analysis of tumor cells were performed using the “monocle” R package (version 2.18.0) (27).

### DEG Identification

After removing the outliers, the expression data were standardized using R software. The “limma” (version 3.46.0) software package (http://www.bioconductor.org/packages/release/bioc/html/limma.html) (28) was used to perform the DEG analysis between the LSCC and adjacent normal control tissues from the GSE142083 dataset. Genes with an expression-adjusted *p* value < 0.05 and log_2_ (fold change) > 1.5 were regarded as significant DEGs. The volcano and heatmap plots were generated using “ComplexHeatmap” and “ggplot2” package. Extracellular matrix-associated genes were annotated using the Matrisome Project (http://matrisomeproject.mit.edu) (29).

### GO Enrichment and KEGG Analysis of DEGs

GO and KEGG enrichment analyses of DEGs and hub modules were performed using the “clusterProfiler” (version 3.18.1) R package (http://www.bioconductor.org/packages/release/bioc/html/clusterProfiler.html) (30). The three main processes in GO analysis were as follows: biological process (BP), molecular function (MF), and cellular component (CC). Statistical significance was set at adjusted-*p* < 0.05. Plots were generated using the “GOplot” (version 1.0.2) package.

### Weighted Gene Co-Expression Network Construction

The “WGCNA” package (version 1.70-3) in R (14) was used to construct co-expression networks of LSCC and adjacent normal tissue samples. Genes with a mean fragments per kilobase per million mapped reads (FPKM) > 0.5 were selected. The adjacency matrices that stored information of the entire co-expression network were created based on Pearson’s correlation matrices. A topological overlap matrix (TOM) was created from the adjacency matrix to estimate the connectivity of the network. Average linkage hierarchical clustering was used to construct a clustering dendrogram of the TOM matrix with a minimum module size of 30. Finally, similar gene modules were merged with a threshold of 0.25.

### Identification and Verification of Prognostic Gene Signatures

The hub genes were identified as intersecting between the midnight-blue, blue, and green-yellow modules from the WGCNA and DEGs in the GSE142083 dataset. These hub genes were validated for their prognostic signature.

First, we identified the LSCC patient cohort through the clinical data from the TCGA-HNSC dataset (TCGA-HNSC-Larynx). Univariate Cox regression analysis of these hub genes was performed to screen the significant genes associated with overall survival (OS) in the training cohort. Genes with *p* < 0.1 were included in the subsequent analysis. Finally, multivariate Cox regression model analysis was performed to establish a prognostic model. The coefficients of multivariate Cox regression analysis were used to calculate the risk score (RS) of each sample using the following formula:


$$RS=\sum_{i=1}^{N}\left(coef_{i}\times expr_{i}\right)$$

According to the RS, samples were divided into a high-risk group and a low-risk group with a cutoff value of 50% in the TCGA-HNSC-Larynx cohort. Receiver operating characteristic (ROC) curve analysis and Kaplan–Meier analysis were conducted between the high-risk and low-risk groups. For ROC analysis, “survivalROC” package (version 1.0.3) was performed to draw 5-year OS ROC curves with the survival status (dead or alive) as outcome variable. Verification was also performed using the GSE27020, GSE39366, and GSE127165 datasets.

### Mutation Analysis

The R package “maftools” (version 2.6.05) was used to analyze the mutation data of TCGA-HNSC-Larynx dataset. The tumor mutation burden (TMB) score for each sample from the high-risk and the low-risk groups was calculated.

### Tumor-Infiltrating Immune Cell Abundance Analysis

The CIBERSORT (https://cibersort.stanford.edu/) algorithm was used to assess the proportions of 22 types of infiltrating immune cells based on the TCGA-HNSC-Larynx dataset and GSE142083 data following a previously reported procedure (31). A bar plot was drawn to show the differences in the composition of 22 kinds of tumor-infiltrating immune cells between the high-risk group and low-risk group.

### Function Analysis of the Prognostic Genes by Single-Gene Gene Set Enrichment Analysis

KEGG analysis was conducted on the high- and low-risk groups *via* Gene Set Variation Analysis (GSVA). The reference information was downloaded from the Molecular Signature Database v7.4 (MSigDB v7.4, http://software.broadinstitute.org/gsea/msigdb/index.jsp) (32). Enriched pathways with *p*-value < 0.05 were considered to be statistically significant.

We conducted single-gene gene set enrichment analysis (GSEA) (33) of the prognostic genes using data from TCGA. All samples were divided into high-expression and low-expression groups based on the median of the prognostic genes, and GSEA was conducted to explore the enrichment of GO biological process (BP) and KEGG pathways in different groups. Statistical significance was set at *p* < 0.05.

### Cell Culture and Transfection

The laryngeal cancer cell line AMC-HN-8 and the dysplastic oral keratinocyte cell line DOK were acquired from the Department of Otolaryngology-Head and Neck Surgery, Xiangya Hospital Central South University. AMC-HN-8 cells were maintained in Dulbecco’s modified Eagle’s medium (DMEM) with high glucose (HyClone, Logan, UT, USA) and 10% fetal bovine serum (FBS) (Biological Industries, CT, USA). DOK cells were cultured in RPMI-1640 medium (HyClone, Logan, Utah, USA) and 10% FBS (Biological Industries, CT, USA). Cells were maintained at 37°C in a humidified incubator with 5% CO_2_.

The *TEDC2* siRNA (siTEDC2 #1 and siTEDC2 #2) was produced by RiboBio Inc. (Guangzhou, China). SiRNA was transfected into cells using Lipofectamine 3000 Reagent (Invitrogen, USA) with Opti-MEM medium (Gibco, USA).

### RNA Isolation and RT-PCR

The total RNA was extracted using TRIzol reagent (Solarbio, Beijing, China) and subjected to reverse transcription with random primers using the RevertAid First Strand cDNA Synthesis Kit (Thermo Scientific, USA). The expression level of targeted genes was measured with Maxima SYBR Green/ROX qPCR Mix (Thermo Scientific, USA) using real-time PCR system (Roche, Basel, Switzerland). The relative RNA expression levels were calculated using the 2^(−△△CT)^ method. The *18 s* rRNA was used as internal controls. The sequences of primers will be provided upon request.

### CCK-8 Assay

For CCK-8 assay, 1,000 cells per well were seeded into 96-well plates. For each well, 10 μl CCK-8 (Solarbio, Beijing, China) was added into the medium. Then, incubated at 37°C for 2 h, the OD value at 450 nm was measured at different time.

### Transwell Assay

For cell migration assay, 2.5 × 10^5^ cells per well that were suspended in DMEM were seeded into a 24-well 8.0-μm transwell top chamber (Jet Biofil, Guangzhou, China). DMEM medium supplemented with 12% FBS was added to the bottom chambers. After incubating at 37°C for 12 h, cells at top chambers were fixed with 4% paraformaldehyde for 30 min, followed by permeabilization with methyl alcohol for 20 min. Then, cells were stained with 0.1% crystal violet (Solarbio, Beijing, China) for 15 min. Cells that did not migrate through the pores were removed by a cotton swab. Cells on the bottom of the chamber were counted using an inverted phase-contrast microscope at low magnifications (×5) (at least three randomly selected fields were quantified).

### Statistical Analysis

Statistical analysis was conducted using R (version 4.0.4) or GraphPad Prism (version 8.0). Student-*t* test was used to compare two normal-distribution groups. NS: not statistically significant; *p < 0.05; **p < 0.01; ***p < 0.001.

## Results

### DEG Screening

**Figure 2:**
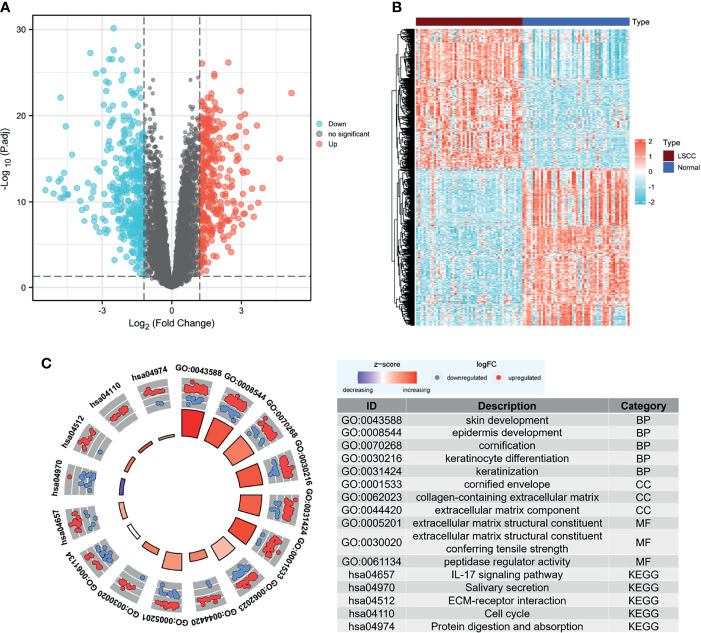
Screening for DEGs. **(A)** Volcano map of DEGs between LSCC and normal samples in the GSE142083 dataset. The red plots in the volcano represent upregulated genes, and the blue points represent downregulated genes. **(B)** Heatmap of all the DEGs. The color in heatmaps from blue to yellow shows the progression from low expression to high expression, respectively. **(C)** GO and KEGG analysis of the DEGs. The outer circle shows the scatter plot of the assigned gene log_2_fold change for all terms: red points show genes that exhibited increased expression, whereas the blue points represent genes that exhibited decreased expression. The inner circle indicates the Z-score value and the number of genes: red represents the higher z-score value, and purple represents a lower Z-score value. DEG, differentially expressed gene; BP, biological process; CC, cell component; MF, molecular function; GO, Gene Ontology; KEGG, Kyoto Encyclopedia of Genes and Genomes; LSCC, laryngeal squamous cell carcinoma.

We analyzed the DEGs in the GSE142083 dataset using the limma package with a threshold of |log_2_(fold-change)| > 1.2 and an adjusted-*p* < 0.05. *A* total of 701 DEGs, including 329 upregulated genes and 372 downregulated genes, were screened between the LSCC and adjacent normal samples. The heatmap and volcano plots show the expression patterns of these DEGs (**Figures 2A, B**).

The clusterProfiler package was then used to determine the role of DEGs in the pathogenesis of LSCC using a cutoff criterion of adjusted-*p* < 0.05. For the biological process group, skin development, epidermis development, cornification, keratinization, and keratinocyte differentiation were significantly enriched. The genes in the cellular component group were significantly enriched in cornified envelope, collagen-containing extracellular matrix (ECM), and ECM component. In the molecular function group, the DEGs were primarily enriched in ECM structural constituent and peptidase regulator activity. KEGG analysis revealed that the IL-17 signaling pathway, salivary secretion, ECM–receptor interaction, cell cycle, and protein digestion and absorption were significantly enriched (**Figure 2C**). We also noticed that ECM-associated genes were enriched in the DEGs, and most of them were highly expressed in the LSCC samples (**Supplementary Figure 2**). Moreover, hsa04970:Salivary secretion was one of the few terms that had decreasing Z-scores. This may indicate a significant decrease in salivary secretion in patients with LSCC.

### Weighted Co-Expression Network Construction and Analysis

**Figure 3:**
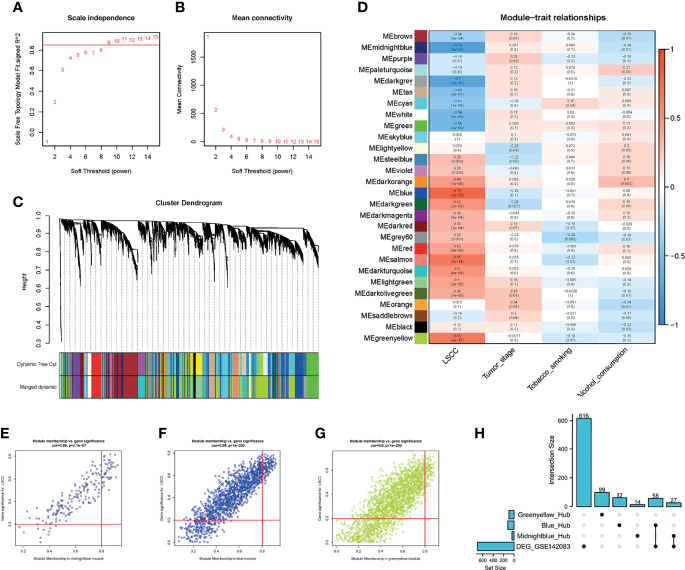
WGCNA of the LSCC samples. **(A, B)** Analysis of the network topology for various soft thresholding powers. **(A)** Scale-free fit index (y-axis) as a function of the soft-thresholding power (x-axis). Horizontal red line shows x = 0.85. **(B)** Mean connectivity (degree, y-axis) as a function of the soft-thresholding power (x-axis). The power was set as 9 for further analysis. **(C)** Hierarchical cluster analysis was performed to detect co-expression clusters with corresponding color assignments. Each color represents a module in the constructed gene co-expression network by WGCNA. **(D)** Module–trait relationships. Each row represents a color module, and every column represents a clinical trait. Each cell contains the corresponding correlation and *p*-value. **(E–G)** A scatter plot of GS for LSCC versus the MM in the **(E)** midnight blue module, **(F)** blue module, and **(G)** green-yellow module. **(H)** UpSet plot for the DEGs in GSE142083 and hub genes from WGCNA. Vertical red line shows the |*MM*| = 0.8; horizontal red line shows the |*GS*| = 0.2. WGCNA, weighted gene co-expression network analysis; LSCC, lung squamous cell carcinoma; GS, gene significance; MM, module membership.

To detect the functional module in LSCC, we applied the WGCNA package based on the GSE142083 dataset to establish the gene co-expression networks. To ensure that the network was scale-free, an empirical analysis was conducted to choose an optimal parameter *β*. Both the scale-free topology model fit index (R^2^) and mean connectivity reached a steady state when *β* = 9 (**Figures 3A, B**).

A total of 28 modules were identified *via* average linkage hierarchical clustering, and each module is represented using a different color in **Figure 3C**. Among the modules, the modules midnight-blue, blue, and green-yellow exhibited a strong correlation with cancer traits (cor ≥ 0.8, *p*-value < 0.001) (**Figures 3D**–**G**). Therefore, these modules were selected as clinically significant modules for further analysis. A set of 400 selected genes was identified for the network heatmap (**Supplementary Figure 3**).

GO and KEGG analyses were used to assess the biological processes, molecular functions, cellular components, and KEGG pathways of genes in the modules. The midnight-blue module was primarily related to glycosylation events and glycosphingolipid biosynthesis, whereas the blue module was primarily associated with the DNA replication and the cell cycle pathway. The green-yellow module was associated with RNA splicing (**Supplementary Figure 4**).

### Identification of the Key Genes

Hub genes were screened using a |module membership (MM) score| > 0.8 and |gene significance (GS) score| > 0.2 as the cutoff criteria (34–36). Based on this criterion, we subsequently sorted the genes according to their connectivity to select candidate genes (**Figures 3E**–**G**). A total of 41 genes in the midnight-blue module, 120 genes in the blue module, and 99 genes in the green-yellow module were screened out.

To identify the “real” key genes, we compared these candidate genes obtained from WGCNA with the 701 DEGs. A total of 85 key genes from the midnight-blue module, blue module, and DEGs were identified (**Figure 3H**).

### Validation of Key Genes *via* Survival Analysis

To validate and explore the prognostic values of these “real” key genes in LSCC, we used the TCGA-HNSC-Larynx dataset (*N* = 111) as training cohort. Univariate Cox regression analysis of the 85 key genes was conducted with regard to the OS of samples from the training cohort (**Supplementary Table 1**). Six genes with *p* < 0.1 were then included for multivariate Cox regression model analysis to establish a prognostic model. Finally, we identified three genes—*SLC35C1*, *HOXB7*, and *TEDC2*—as potential independent prognostic markers for LSCC (**Supplementary Figure 5**). The risk score for each patient was calculated using the following formula: risk score = 0.703437*SLC35C1 + 0.833199*HOXB7 + (-0.891338)*TEDC2.

**Figure 4:**
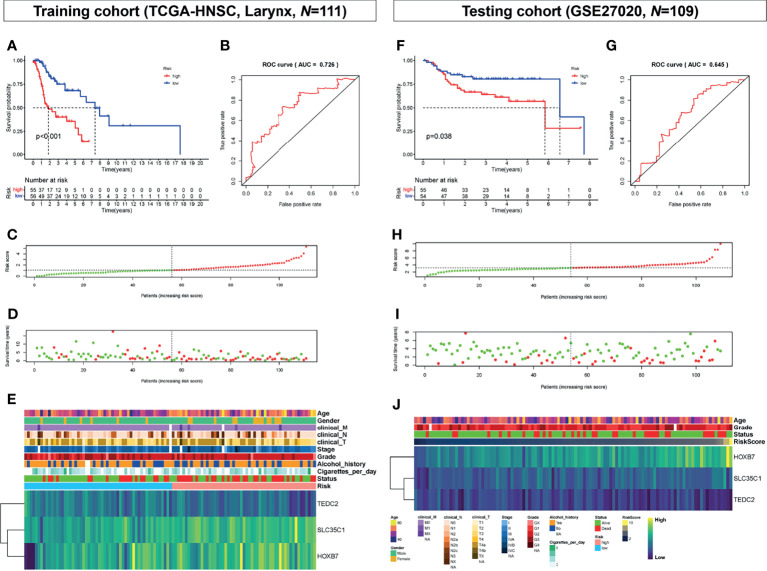
Prognostic analysis of the key genes. **(A)** Overall survival between high-risk score and low-risk score groups in the training cohort (TCGA-HNSC, larynx, *N* = 111). **(B)** ROC curve analysis between high-risk score and low-risk score groups in the training cohort. **(C)** Risk score distribution of patients in the training cohort. **(D)** Survival status scatter plots for patients in the training cohort. **(E)** Expression patterns of risk genes in the training cohort. **(F)** Overall survival between high-risk score and low-risk score groups in the testing cohort (GSE27020, *N* = 109). **(G)** ROC curve analysis between high-risk score and low-risk score groups in the testing cohort. **(H)** Risk score distribution of patients in the testing cohort. **(I)** Survival status scatter plots for patients in the testing cohort. **(J)** Expression patterns of risk genes in the testing cohort. ROC, receiver operating characteristic; AUC, area under the curve.

According to the level of risk score, samples from the two cohorts were divided into a high-risk group and a low-risk group based on a cutoff value of 50% in both training and testing cohorts, as shown in **Figures 4A**–**G**. The risk score was significantly associated with the survival time of patients with LSCC in the training cohort (TCGA-HNSC-Larynx) (**Figure 4A**) and testing cohort (GSE27020) (**Figure 4F**). The ROC curve was then used to evaluate the accuracy of the survival analysis. In the training and testing cohorts, the area under the curve (AUC) was 0.726 and 0.645, respectively, indicating that the prediction effect was good (**Figures 4B, G**). Besides, patients with continuous risk scores harbored various clinical outcomes in different groups (**Figures 4C, D, H**, **I**). Additionally, all three genes (*SLC35C1*, *HOXB7*, and *TEDC2*) were significantly associated with poor prognosis and unhealthy living habits, in either the training cohort (TCGA-HNSC-Larynx), the testing cohort (GSE27020), or other LSCC datasets (GSE39366 and GSE127165) (**Figures 4E, J** and **Supplementary Figure 6**).

### Gene Mutation in the Different Risk Groups

**Figure 5:**
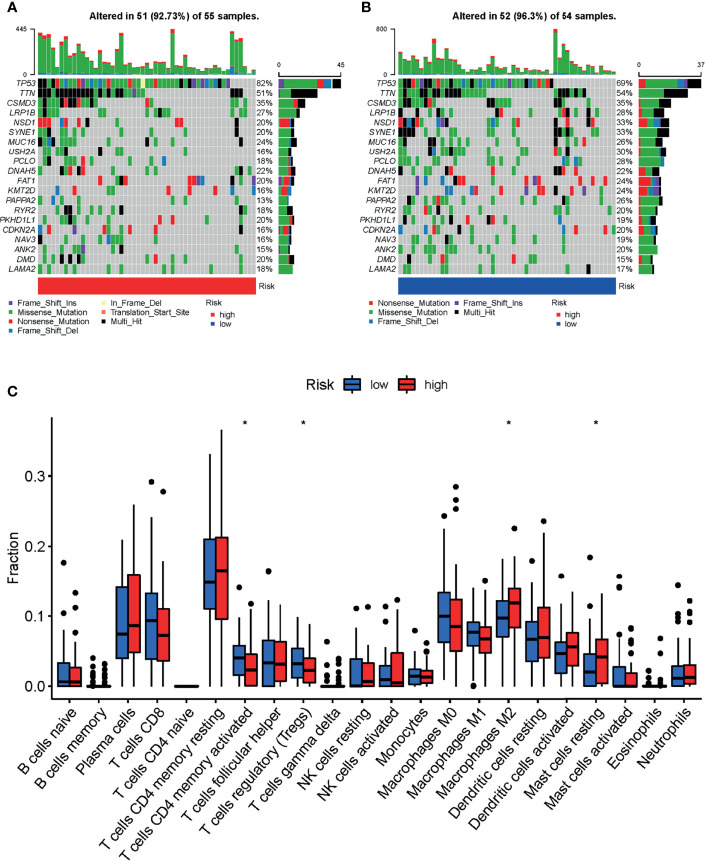
Landscape of mutation profiles and tumor-infiltrating immune cells abundance between high and low-risk LSCC patients. Waterfall plots represent mutation information in each sample of the **(A)** high- and **(B)** low-risk group of TCGA-HNSC-Larynx dataset. **(C)** The difference of 22 tumor-infiltrating immune cells between the high- and low-risk group of TCGA-HNSC-Larynx dataset. *p < 0.05; LSCC, lung squamous cell carcinoma.

Analysis of differences in gene mutations between high-risk and low-risk groups showed that the proportion of patients with gene mutations was 92.73% (51 in 55) and 96.30% (52 in 54) in the high-risk and low-risk score groups, respectively (**Figures 5A, B**). The mutation frequency of *TP53* was observably higher in the high-risk group (82%) than in the low-risk group (69%) (**Figures 5A, B**).

### Comparison of Tumor-Infiltrating Immune Cell Abundance Between Different Risk Groups

The distribution of tumor-infiltrating immune cells is an important indicator of a patient’s lymph node status and prognosis. In order to explore the relationship of the prognostic model and the tumor immune microenvironment, we analyzed the tumor-infiltrating immune cell abundance in the TCGA-HNSC-Larynx dataset using the CIBERSORT algorithm (**Figure 5C**). The results showed that the infiltration levels of the following cells: “T cells CD4 memory activated”, and “T cells regulatory” were significantly higher in the low-risk group than in the high-risk group. In contrast, the infiltration levels of “Macrophages M2” and “Mast cells resting” were significantly lower in the low-risk group. A similar abundance was also estimated in the GSE27020 dataset (**Supplementary Figure 7**).

### Functional Enrichment of Prognostic Genes

**Figure 6:**
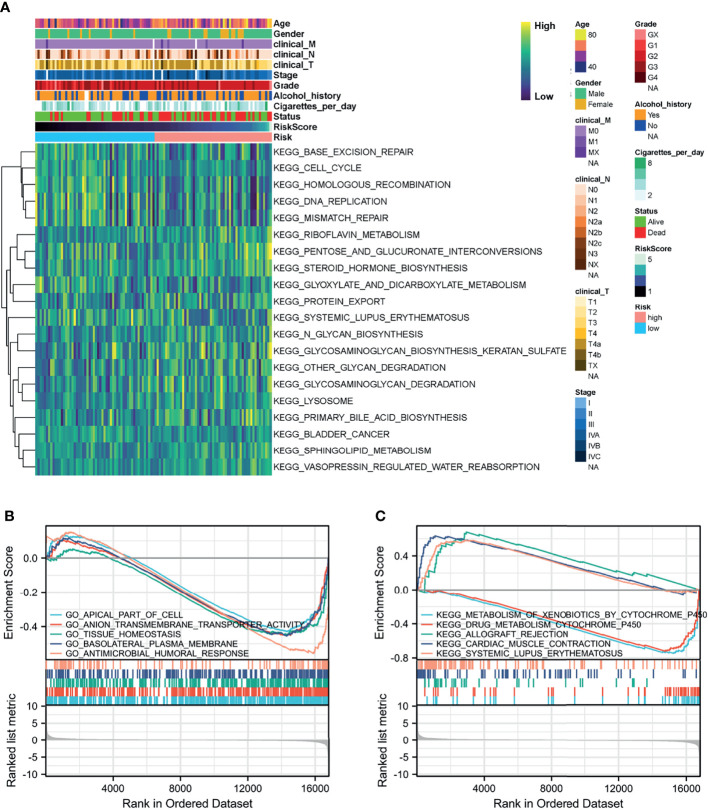
Function analysis of the prognostic model. **(A)** Heatmap of KEGG analysis based on risk score in the TCGA-HNSC-Larynx dataset. **(B, C)** GSEA analysis for GO and KEGG enrichment in the TCGA-HNSC-Larynx dataset according to risk score. GSEA, gene set enrichment analysis; KEGG, Kyoto Encyclopedia of Genes and Genomes; GO, Gene Ontology.

GSVA and GSEA were conducted to search for GO and KEGG pathways in which the prognostic genes or risk scores were enriched in samples with high-risk levels from the TCGA-HNSC-Larynx dataset. Metabolism and cancer-associated pathways including “Glyoxylate and dicarboxylate metabolism”, “Protein export”, “Sphingolipid metabolism”, “Lysosome”, and “Bladder cancer” were significantly enriched with low expression of most prognostic genes and high-risk score (**Figure 6A**). Besides, GSEA based on DEGs between the high- and low-risk score groups also revealed that “Apical part of cell”, “Anion transmembrane transporter activity”, and “Tissue homeostasis” were significantly enriched with GO BP (**Figure 6B**) and “Metabolism of xenobiotics by cytochrome p450”, “Cardiac muscle contraction”, and “Systemic lupus erythematosus” were significantly enriched with KEGG analysis (**Figure 6C**).

### Single-Cell Transcriptomic Context of the Prognostic Genes

**Figure 7:**
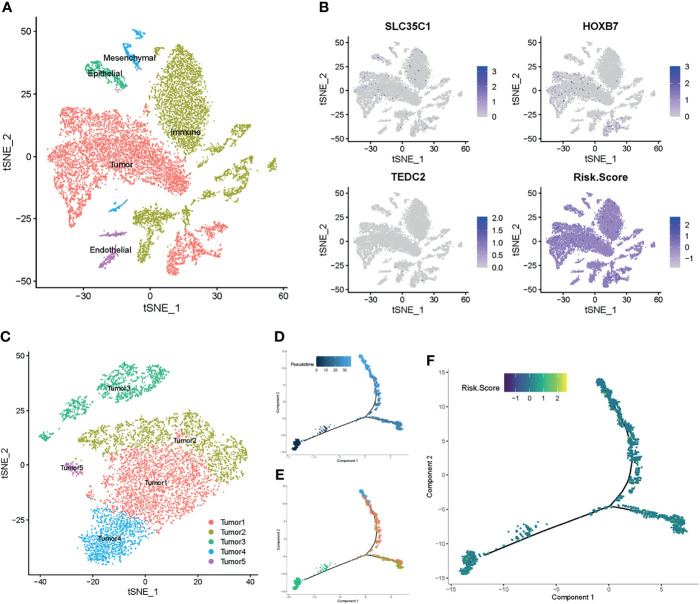
Prognostic expression profile based on single-cell sequencing analysis. **(A)** Composition and distribution of single cells from GSE150321. **(B)** The distribution profile of *SLC35C1*, *HOXB7*, *TEDC2*, and risk score for each cell by the t-SNE plot. **(C)** t-SNE embedding of the tumor cells. **(D, E)** Pseudotime and trajectory analysis revealed the tendency curve from tumor 3 cluster to tumor 4 and tumor 1 clusters. Y-axis means the value of principal component 1 (the first principal direction of maximum sample change), and X-axis means the value of principal component 2 (the second principal direction of maximum sample change). **(F)** The expression profile of risk score annotated in pseudotime and trajectory plot. t-SNE, t-distributed stochastic neighbor embedding.

To further verify the relationship between the prognostic genes and risk score in LSCC, we employed single-cell RNA-Seq data from the GSE150321 dataset (25), which comprised data from two LSCC samples. We identified five cell groups, namely tumor cells, immune cells, epithelial cells, mesenchymal cells and endothelial cells (**Figure 7A**). We then calculated the risk score for each cell and plotted them in a t-SNE plot and the violin plots (**Figure 7B** and **Supplementary Figure 8**). Tumor cells and some immune cells showed higher risk scores than other cell types.

Next, we profiled the tumor cells and arranged them into five clusters (**Figure 7C** and **Supplementary Figures 9A, B**). Pseudotime and trajectory analyses of the tumor cells suggested a continuous cell fate, starting at tumor 3 and progressing toward tumor 1 and tumor 4, with tumor 2 and tumor 5 being a transitioning state (**Figures 7D, E**). Diffusion mapping placed tumor 4 and tumor 1 within populations of high-risk score cells, indicating a differentiation trajectory from low-risk tumor cells to high-risk tumor cells (**Figure 7F**). Together with previous results, these results further supported that the identified prognostic genes influence the LSCC progression.

### TEDC2 Affects Tumor Cell Proliferation and Migration

**Figure 8:**
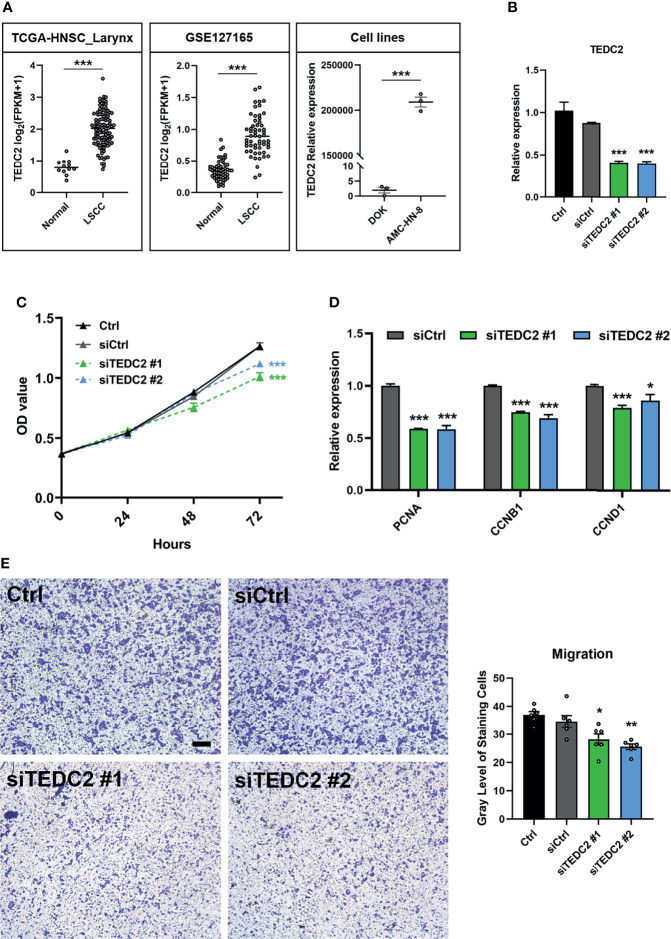
*TEDC2* affects tumor cell proliferation and migration. **(A)** Expression profile of *TEDC2* between normal and LSCC samples in the TCGA-HNSC-Larynx dataset (left) and GSE127165 (middle). *TEDC2* expression between laryngeal cancer cell line AMC-HN-8 and dysplastic oral keratinocyte cell line DOK (right). **(B)** AMC-HN-8 cells were transfected with siRNAs targeting *TEDC2* (siTEDC2 #1 and siTEDC2 #2) or normal control siRNAs (siCtrl) for 48 h. The expression of *TEDC2* was examined by RT-PCR. **(C)** AMC-HN-8 cells were transfected with siRNAs targeting *TEDC2* (siTEDC2 #1 and siTEDC2 #2) or normal control siRNAs (siCtrl), then cell proliferation was determined by CCK-8 assay. **(D)** Expressions of *PCNA*, *CCNB1*, and *CCND1* were examined by RT-PCR. **(E)** The migration abilities of AMC-HN-8 cells after transfected with siRNAs targeting *TEDC2* (siTEDC2 #1 and siTEDC2 #2) or normal control siRNAs (siCtrl) were detected by transwell assays. Bar, 200 μm. Error bars represent SEM of at least three independent experiments; *p < 0.05; **p < 0.01; ***p < 0.001.

Based on the results of the previous analysis, we selected *TEDC2*, one of the three prognostic genes with less research involving LSCC, for further analysis. We first verified the expression level of *TEDC2* in different datasets, and they both indicated that *TEDC2* was higher in LSCC than in paired normal tissues (**Figure 8A**). Similar results were observed in the LSCC cell line and a precancerous cell line (**Figure 8A**). We therefore used siRNA to inhibit the expression of *TEDC2*. Results from RT-PCR indicated that both of the two *TEDC2* siRNAs in AMC-HN-8 showed a good silenced efficiency (**Figure 8B**).

CCK-8 assays showed that silencing the expression of *TEDC2* promoted AMC-HN-8 cell proliferation (**Figure 8C**). Cell-cycle genes, including *PCNA*, *CNNB1*, and *CNND1*, were both downregulated when *TEDC2* was knocked down (**Figure 8D**). Results of the transwell assays showed that knockdown of *TEDC2* promoted migration (**Figure 8E**). Based on these validations, we concluded that silenced *TEDC2*, to a certain extent, promoted LSCC cell proliferation and migration.

## Discussion

In the present study, 701 DEGs in the GSE142083 dataset were screened. We firstly constructed weighted co-expression networks using the WGCNA algorithm based on the GSE142083 dataset. Three modules in LSCC tissues were detected based on the co-expression network. After intersecting with the DEGs, 85 key genes were screened out. Following univariate and multivariate analyses, a novel prognostic model for LSCC based on three genes (*SLC35C1*, *HOXB7*, and *TEDC2*) was established.

LSCC is a leading malignant type of HNSCC. There is an urgent need to identify potential targets for drugs and biomarkers to improve early diagnosis and outcomes (37–40). A growing body of evidence suggests that solid tumor was stiffer than normal tissue (41). Moreover, the increased deposition of ECM proteins is one of the main reasons for this. The ECM is a fundamental and important component of all tissue organs; it also interacts with tumor cells and regulates tumor growth, proliferation, differentiation, adhesion, and metastasis (42–44). Here, we confirmed that most of the core ECM genes, such as collagens, proteoglycans, and glycoproteins, were highly expressed in the LSCC tissues than in the adjacent normal mucosal tissues (**Supplementary Figure 2**). Moreover, ECM-associated biological processes were enriched in the DEGs of the GSE142083 dataset (**Figure 2C**). These results further confirmed the importance of ECM accumulation in LSCC. Due to the excess deposition of ECM, tumors in this region may be regarded as difficult to treat (45–47). Therefore, exploring novel methods to reduce ECM deposition may be a strategy for LSCC treatment.

SLC35C1, as a member of the solute carrier (SLC) family, encoded a guanosine 5′-diphosphate (GDP)-fucose transporter 1 channel that critically regulated the fucosylation of glycans. Mutation of *SLC35C1* was found to cause leukocyte adhesion deficiency, which was a rare congenital disease due to the defect in the biosynthesis of selectin ligands on leukocytes (48). Fucosylation has been found to be an important type of posttranslational modification in many types of cancer (49). Moriwaki et al. have reported that *SLC35C1*, as a GDP-fucose transporter, was highly expressed and with an increased level of fucosylation in hepatocellular carcinoma (50). In colon cancer, however, Deng et al. showed that *SLC35C1* was reduced in all colon cancers and they further proved that loss of *SLC35C1* may promote colon cancer progression through the activation of the Wnt signaling pathway (51). In our analysis, we firstly identified that *SLC35C1* might be a potential independent prognostic marker for LSCC and the upregulation of *SLC35C1* may have an association with high risk score and poor prognosis of LSCC patient (**Figures 4E, J**). Nevertheless, future studies are warranted to clarify the underlying mechanisms of *SLC35C1* and fucosylation in LSCC.

Homeobox B7 (*HOXB7*) is a member of the Antp homeobox family and encodes a protein with a homeobox DNA-binding domain. As a sequence-specific transcription factor, HOXB7 has been shown to have specific functions in cellular proliferation, differentiation, and death (52). To date, *HOXB7* has been implicated to be aberrantly expressed in several types of cancers, including breast cancer (53), gliomas (54), gastric cancer (55), esophageal squamous cell carcinoma (56), intrahepatic cholangiocarcinoma (57), and cervical cancer (58). Furthermore, study from Wu et al. (59) and study from Mo et al. (60) both proved that *HOXB7* may serve as an oncogene for HNSCC and LSCC. Our analysis of TCGA-HNSC-Larynx, GSE27020, GSE39366, and GSE127165 further confirmed the role of the potential prognostic marker of HOXB7 in LSCC and HNSCC. However, its detailed mechanism still requires further research.

In comparison with the other two prognostic genes, *TEDC2* has not been intensively investigated in cancer. *TEDC2* is also called as tubulin epsilon and delta complex 2 or *C16ORF59*. As the name implies, TEDC2 is a component of the cytoskeleton. A study from Hsu et al. showed that *TEDC2* was differentially expression between lung adenocarcinoma and paired normal tissues (61). Recently, Meng et al. used the monozygotic twin-pair database to detect the alteration of DNA methylation after alcohol drinking, and they found that a hypermethylation of cg07326074, located in *TEDC2*, was associated with alcohol consumption (62). This drinking-related methylation was also suspected to affect *TEDC2* gene function. Alcohol consumption is an established risk factor for HNSC (63). In TCGA-HNSC, we also confirmed that the expression of *TEDC2* was increased in the alcohol consumption cohort (**Supplementary Figure 10**), and knocking down *TEDC2* could inhibit the capacities of proliferation and migration in the LSCC cell line (**Figures 8A**–**E**). Therefore, as a tumor-promoting gene, *TEDC2* may increase in the condition of alcohol consumption and thus promote tumorigenesis and metastasis. In the future, detailed mechanism research still needed to elucidate the association of *TEDC2* and tumor.

## Conclusion

Following the screening of DEGs, and WGCNA of LSCC and adjacent normal tissues, a novel prognostic model based on three genes (*SLC35C1*, *HOXB7*, and *TEDC2*) for LSCC was identified. These prognostic genes may play a significant role in improving the prognostic prediction of patients with LSCC and may also serve as therapeutic targets and/or biomarkers for LSCC.

## Data Availability Statement

The datasets presented in this study can be found in online repositories. The names of the repository/repositories and accession number(s) can be found in the article/**Supplementary Material**.

## Author Contributions

HH and AP conceived and directed the project. CH and YD collected the data and information. CH, LH, JH, and YC analyzed and interpreted the data. CH and HH wrote the manuscript with the help of all the other authors. All authors contributed to the article and approved the submitted version.

## Funding

This study was supported by the Project funded by the China Postdoctoral Science Foundation (2020TQ0363 and 2020M682598); the National Natural Science Foundation of China (81570928); the Natural Science Foundation of Hunan, China (2021JJ40992); and the Fundamental Research Funds for the Central Universities of Central South University (2021zzts0078).

## Conflict of Interest

The authors declare that the research was conducted in the absence of any commercial or financial relationships that could be construed as a potential conflict of interest.

## Publisher’s Note

All claims expressed in this article are solely those of the authors and do not necessarily represent those of their affiliated organizations, or those of the publisher, the editors and the reviewers. Any product that may be evaluated in this article, or claim that may be made by its manufacturer, is not guaranteed or endorsed by the publisher.
