# Supplementary material for: Identification of Novel Prognostic Markers Associated With Laryngeal Squamous Cell Carcinoma Using Comprehensive Analysis
Source: Front Oncol. 2022 Jan 11;11:779153. doi: 10.3389/fonc.2021.779153 (PMC8787159; doi:10.3389/fonc.2021.779153)
Supplement: Supplementary file 1 [file DataSheet_1.docx]

Supplementary Material

# Supplementary Figures and Tables

## Supplementary Figures


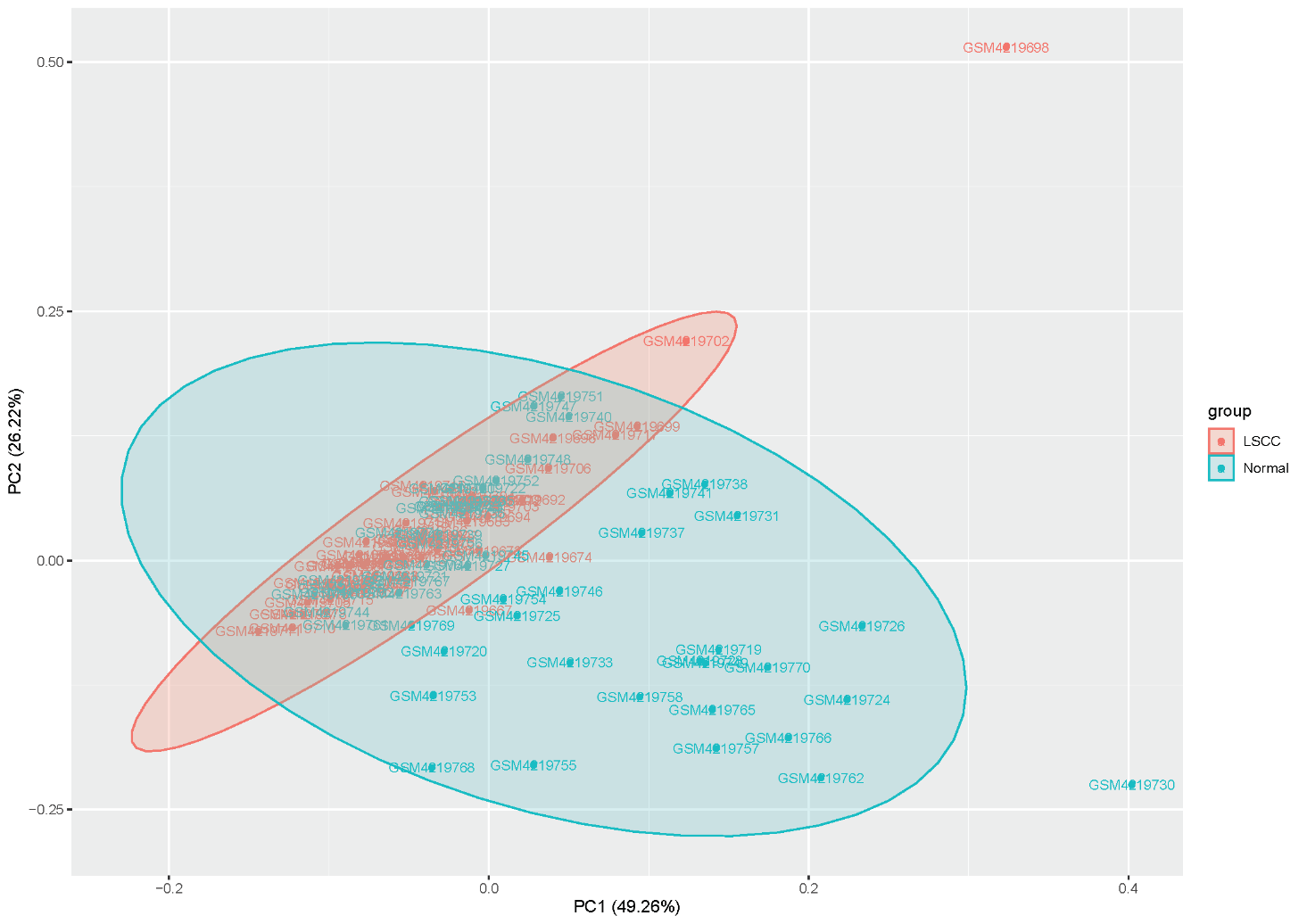


**Supplementary Figure 1.** PCA based on the whole gene list. PCA, principal component analysis.


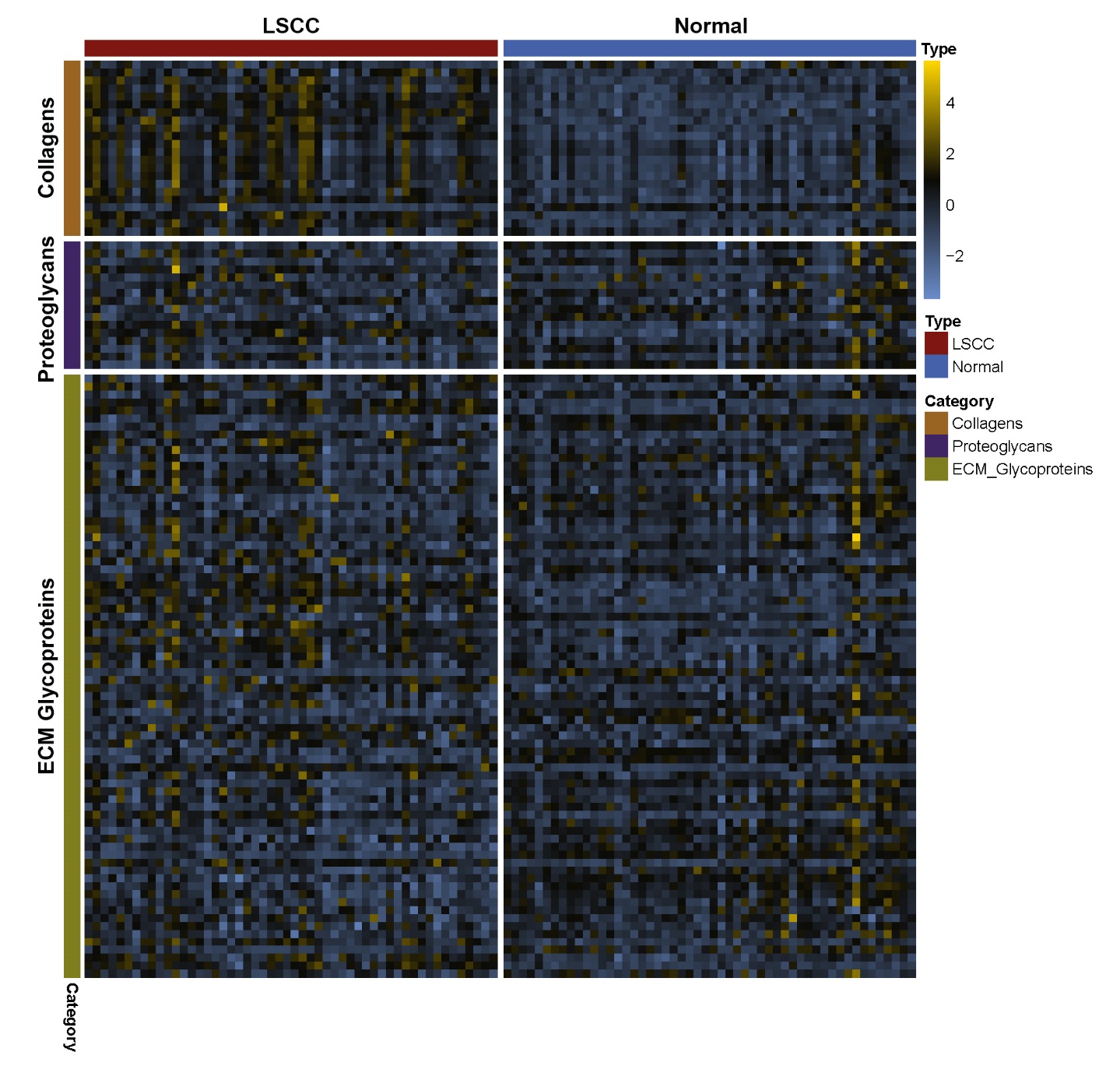


**Supplementary Figure 2.** Heatmap of matrisome genes in the GSE142083 dataset.


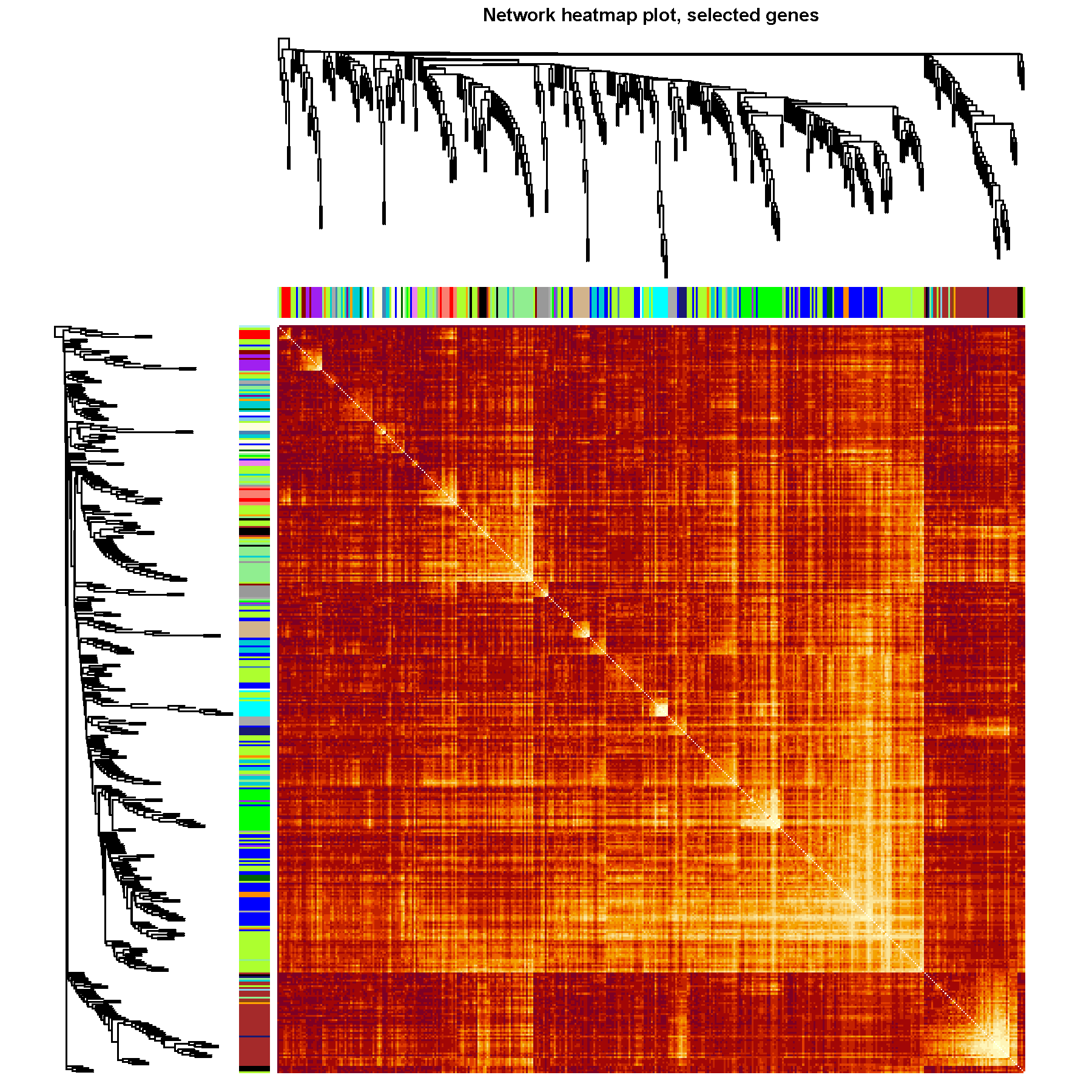


**Supplementary Figure 3.** Heatmap depicting the TOM of genes selected for weighted co-expression network analysis. Light color represents lower overlap, and red represents higher overlap. TOM, Topological Overlap Matrix.


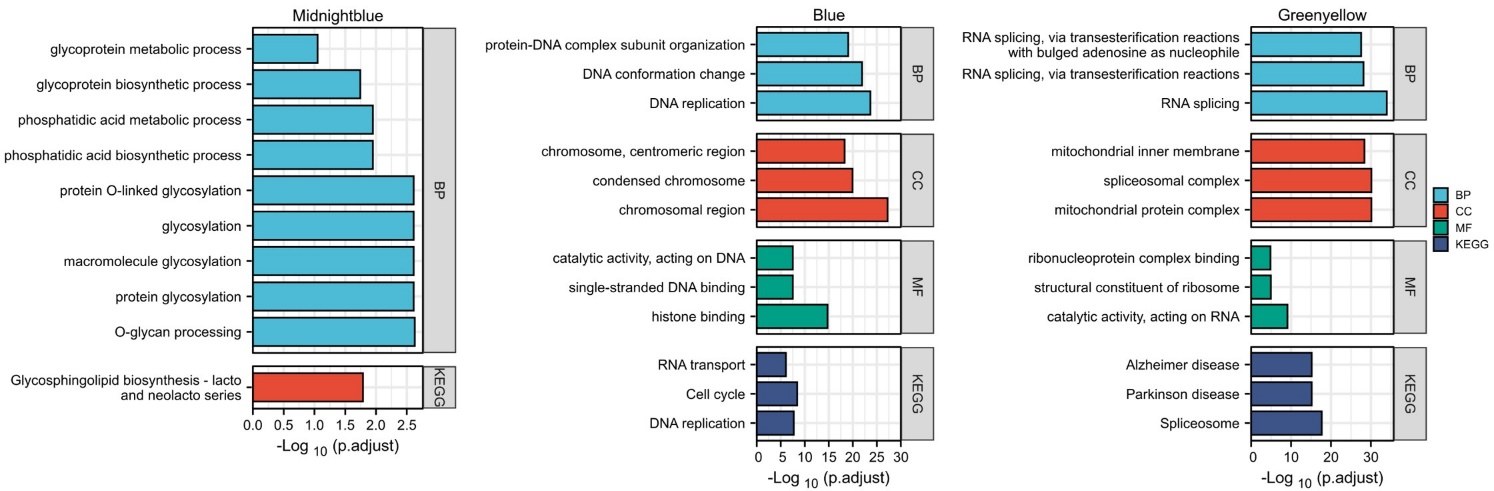


**Supplementary Figure 4.** GO and KEGG analysis of the midnight blue, blue, and greenyellow module. BP, biological process; CC, cell component; MF, molecular function; GO, Gene Ontology; KEGG, Kyoto Encyclopedia of Genes and Genomes.


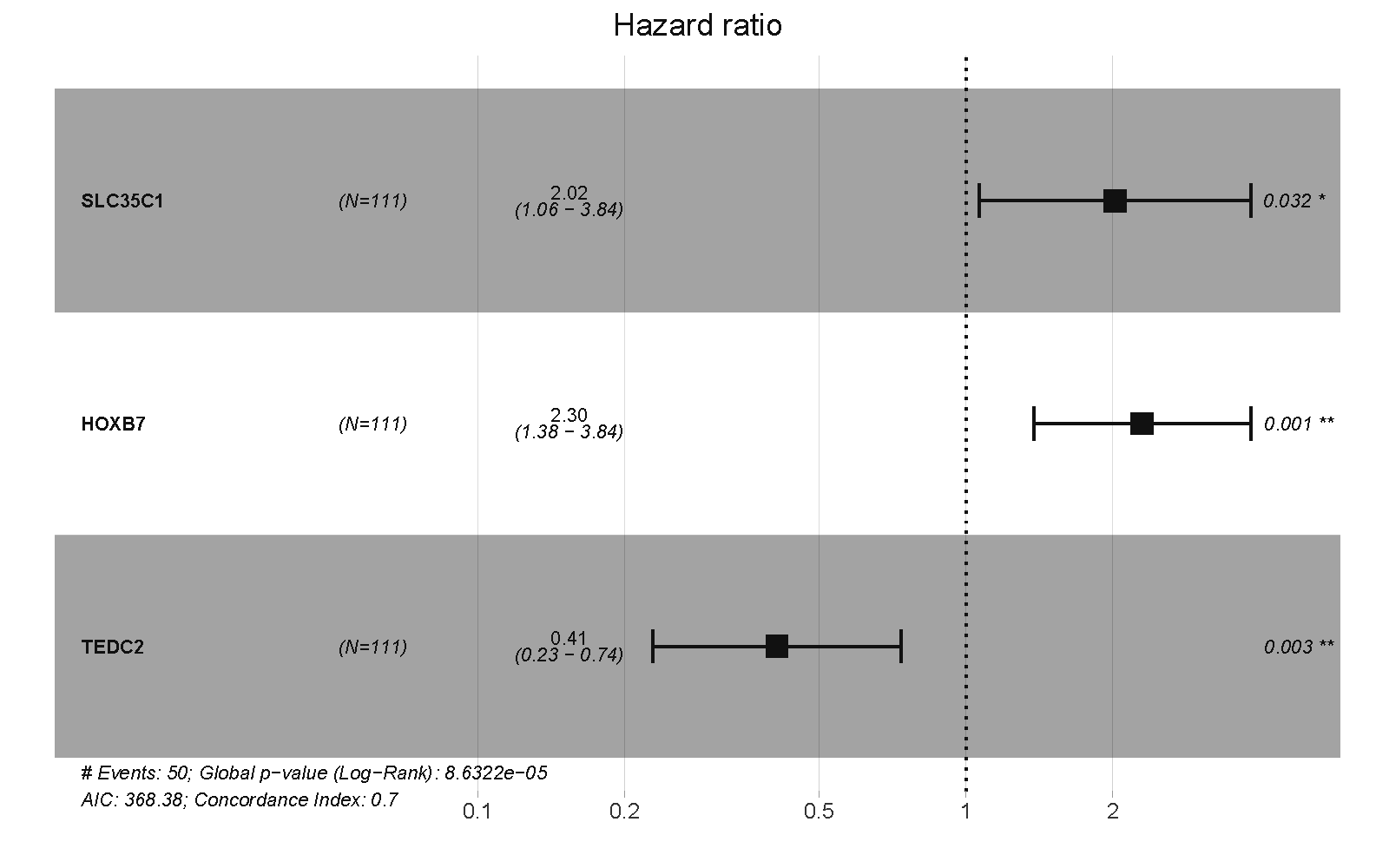


**Supplementary Figure 5.** Forest maps of prognosis-related genes in the training cohort by multivariate Cox regression analysis.


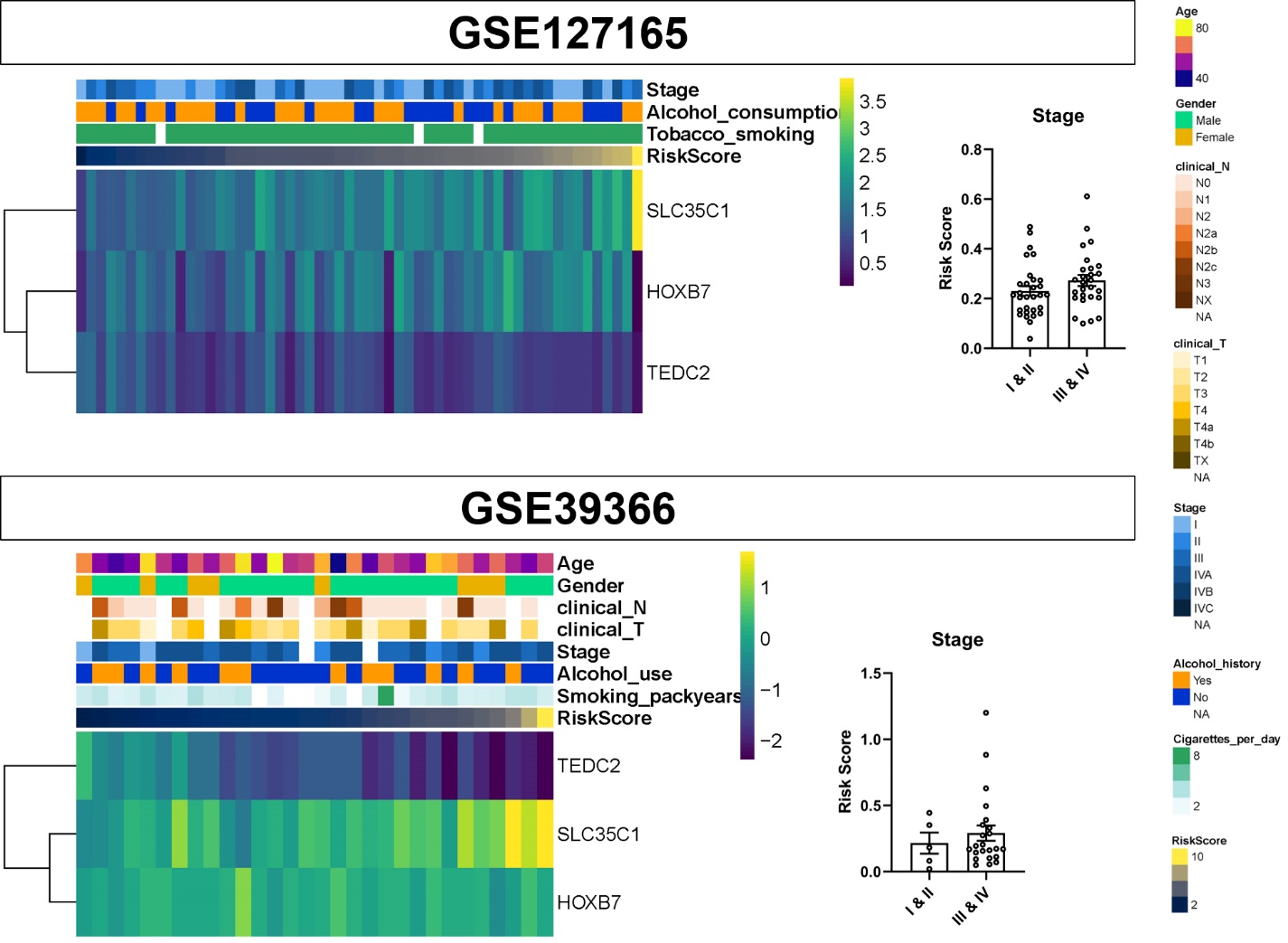


**Supplementary Figure 6.** Expression patterns of risk genes in the GSE127165 and GSE39366 datasets.


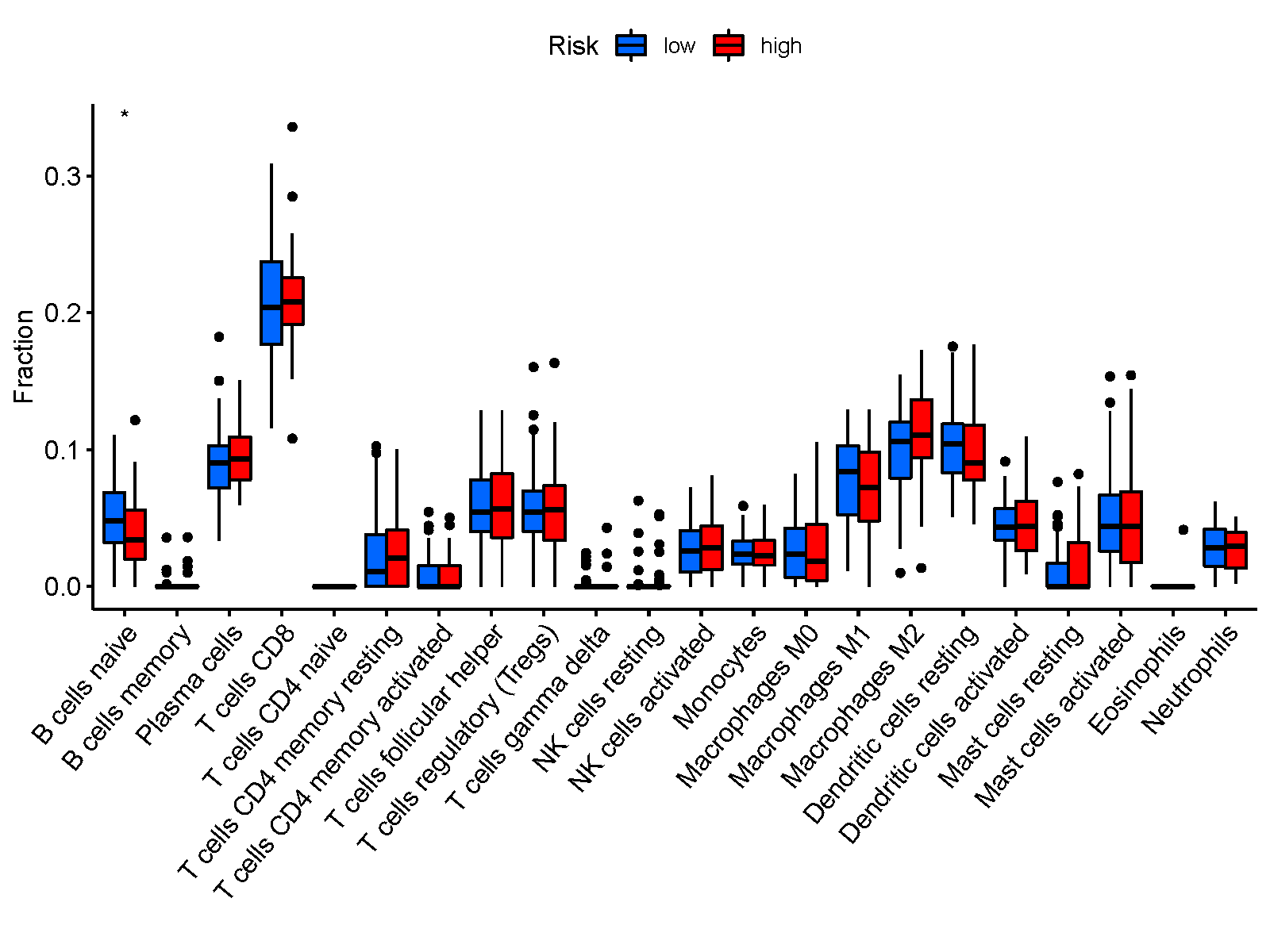


**Supplementary Figure 7.** The differences in the 22 tumor-infiltrating immune cells between the high- and low-risk group of GSE27020 dataset.


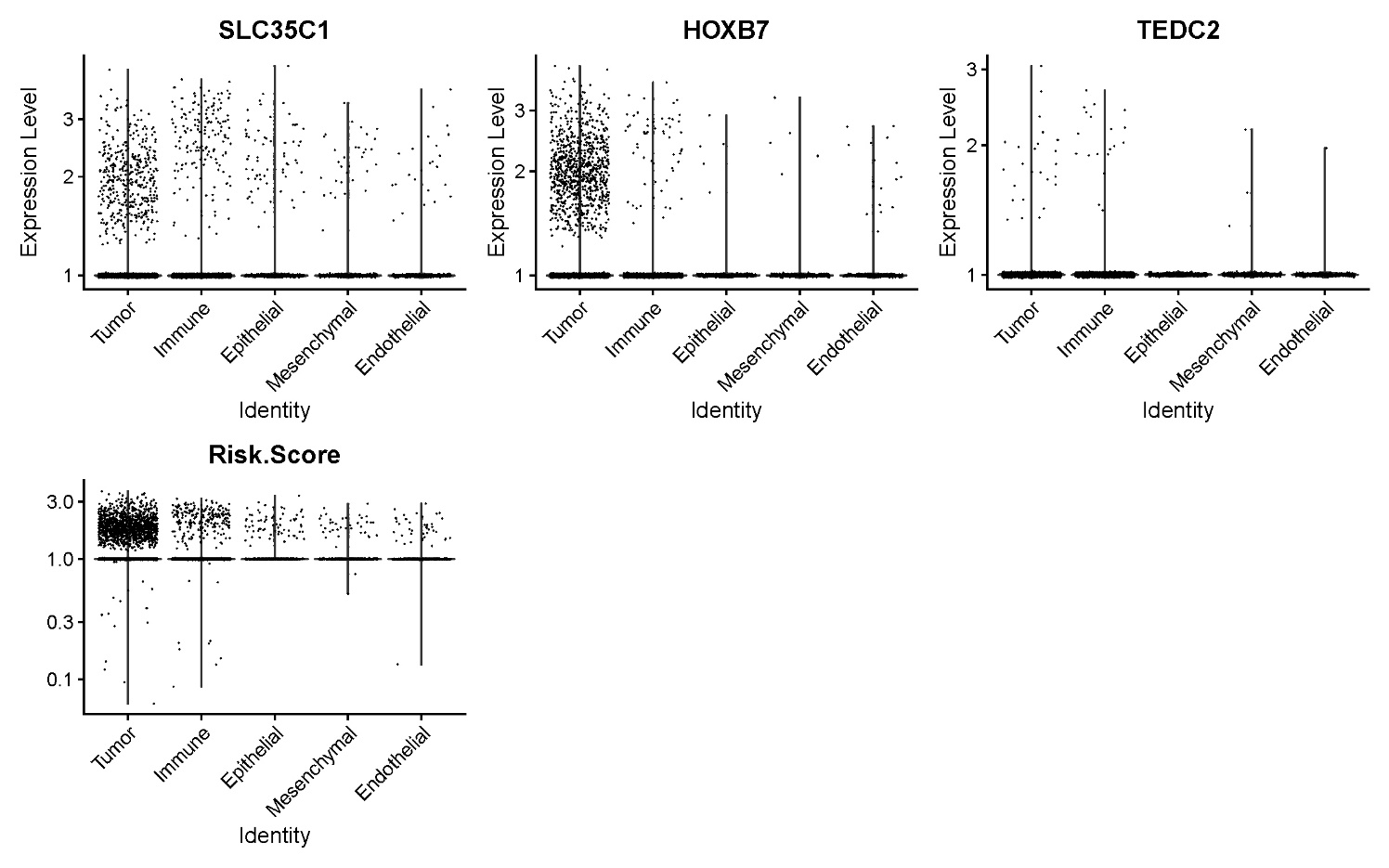


**Supplementary Figure 8.** Expression profiles of *SLC35C1*, *HOXB7*, *TEDC2* and risk score by violin plots.

**
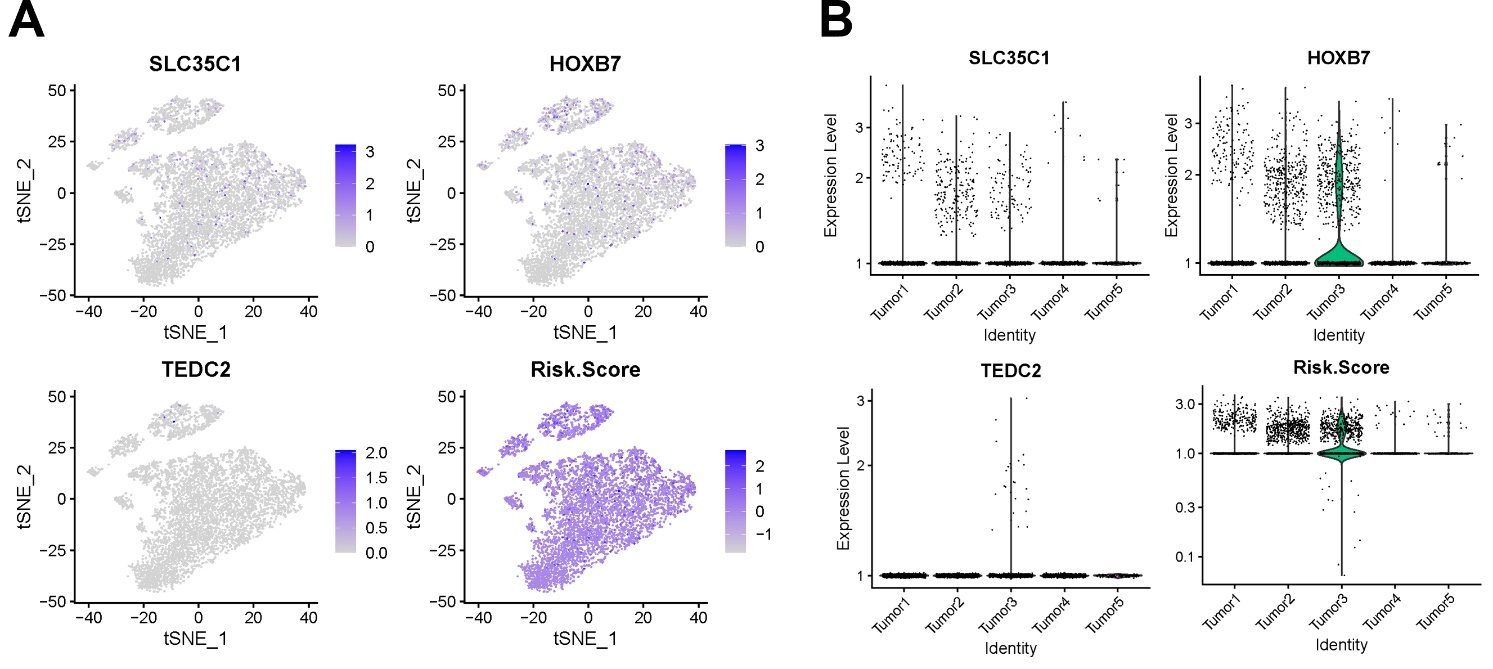
**

**Supplementary Figure 9.** Expression profiles of *SLC35C1*, *HOXB7*, *TEDC2* and risk score by the **(A)** t-SNE plots **(B)** violin plots in tumor cell clusters.


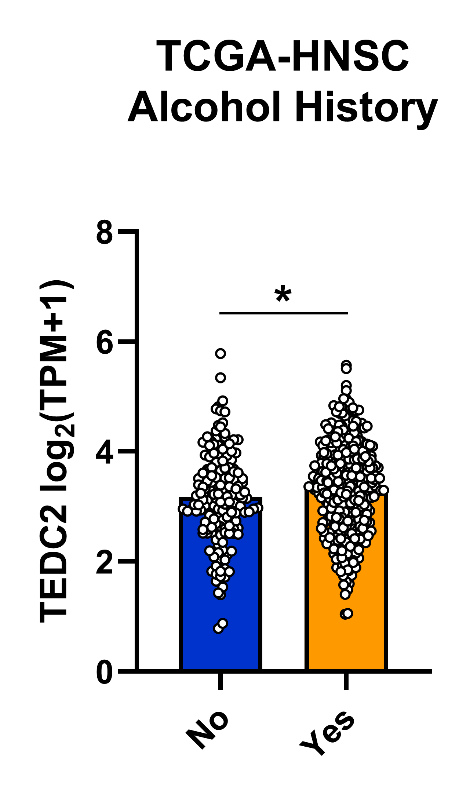


**Supplementary Figure 10.** Expression of *TEDC2* between different alcohol history patients in TCGA-HNSC dataset. **p* < 0.05.

## Supplementary Tables

**Supplementary Table 1. Univariate Cox regression analysis in the train cohort**

| **Characteristics** | **HR** | **95% CI** | ***p*-value** |
| --- | --- | --- | --- |
| *CENPE* | 0.835 | 0.462-1.507 | 0.549 |
| *NEK2* | 0.987 | 0.597-1.629 | 0.958 |
| *DLGAP5* | 0.942 | 0.577-1.540 | 0.813 |
| *TTK* | 1.201 | 0.722-1.996 | 0.481 |
| *B3GNT3* | 0.946 | 0.724-1.236 | 0.685 |
| *TOP2A* | 1.057 | 0.748-1.495 | 0.752 |
| *DEPDC1* | 1.208 | 0.684-2.135 | 0.515 |
| *CENPA* | 0.929 | 0.537-1.608 | 0.793 |
| *GCHFR* | 0.736 | 0.461-1.175 | 0.200 |
| *FAM3B* | 1.014 | 0.824-1.248 | 0.897 |
| *CDC6* | 0.912 | 0.578-1.438 | 0.690 |
| *GALNT12* | 0.927 | 0.649-1.323 | 0.676 |
| *CENPF* | 0.960 | 0.646-1.426 | 0.839 |
| *TPX2* | 1.012 | 0.667-1.535 | 0.955 |
| *CAPN5* | 1.311 | 0.888-1.935 | 0.173 |
| *OMP* | 1.731 | 0.078-38.363 | 0.729 |
| *FEN1* | 1.337 | 0.776-2.305 | 0.295 |
| *H2AC11* | 1.416 | 0.921-2.177 | 0.113 |
| *CLDN7* | 1.007 | 0.797-1.272 | 0.954 |
| *PLK1* | 1.057 | 0.600-1.862 | 0.848 |
| *SLC35C1* | 1.987 | 1.089-3.626 | 0.025 |
| *ZWINT* | 0.838 | 0.519-1.353 | 0.470 |
| *PAX9* | 0.943 | 0.739-1.203 | 0.636 |
| *RRM2* | 1.037 | 0.651-1.654 | 0.877 |
| *MUC1* | 0.997 | 0.803-1.238 | 0.979 |
| *ASPM* | 0.954 | 0.597-1.526 | 0.845 |
| *MAD2L1* | 0.881 | 0.504-1.542 | 0.657 |
| *MYBL2* | 0.881 | 0.579-1.339 | 0.552 |
| *H2BC13* | 0.804 | 0.269-2.402 | 0.697 |
| *KIF2C* | 0.746 | 0.471-1.181 | 0.211 |
| *FANCI* | 0.990 | 0.608-1.612 | 0.967 |
| *ADGRF1* | 1.016 | 0.724-1.426 | 0.925 |
| *RFC4* | 1.016 | 0.640-1.612 | 0.947 |
| *KNL1* | 1.017 | 0.592-1.747 | 0.951 |
| *KIF11* | 0.920 | 0.585-1.447 | 0.718 |
| *ELF3* | 0.938 | 0.764-1.150 | 0.538 |
| *CEACAM6* | 1.037 | 0.883-1.218 | 0.658 |
| *ANLN* | 1.253 | 0.863-1.818 | 0.236 |
| *CENPN* | 1.255 | 0.853-1.847 | 0.248 |
| *UBE2C* | 0.985 | 0.659-1.473 | 0.942 |
| *CDCA5* | 0.879 | 0.525-1.471 | 0.624 |
| *KPNA2* | 1.188 | 0.687-2.055 | 0.538 |
| *FOXM1* | 1.060 | 0.752-1.495 | 0.739 |
| *AURKA* | 1.073 | 0.673-1.710 | 0.767 |
| *TROAP* | 0.841 | 0.531-1.331 | 0.459 |
| *SH3BGRL2* | 1.035 | 0.771-1.389 | 0.821 |
| *RAD51AP1* | 0.999 | 0.653-1.530 | 0.998 |
| *ARHGAP11A* | 1.079 | 0.629-1.850 | 0.783 |
| *CCNB2* | 0.990 | 0.562-1.742 | 0.971 |
| *KIF23* | 1.153 | 0.675-1.970 | 0.601 |
| *NUSAP1* | 0.964 | 0.638-1.458 | 0.863 |
| *HOXB7* | 1.570 | 1.083-2.276 | 0.017 |
| *CDK1* | 0.953 | 0.579-1.568 | 0.849 |
| *GINS2* | 1.183 | 0.707-1.978 | 0.522 |
| *TRNP1* | 1.112 | 0.868-1.425 | 0.401 |
| *CDCA8* | 0.968 | 0.615-1.523 | 0.888 |
| *FUT2* | 0.823 | 0.600-1.130 | 0.229 |
| *KIFC1* | 0.732 | 0.452-1.185 | 0.204 |
| *TJP3* | 0.787 | 0.593-1.045 | 0.098 |
| *CEP55* | 1.043 | 0.689-1.578 | 0.844 |
| *BUB1* | 0.846 | 0.510-1.404 | 0.518 |
| *UBE2T* | 0.969 | 0.626-1.501 | 0.888 |
| *CYP3A5* | 1.025 | 0.658-1.595 | 0.914 |
| *IQGAP3* | 0.904 | 0.565-1.446 | 0.675 |
| *KIF18B* | 0.730 | 0.458-1.164 | 0.186 |
| *PCDH1* | 1.014 | 0.695-1.479 | 0.944 |
| *GPD1L* | 0.874 | 0.571-1.340 | 0.537 |
| *SYNGR1* | 1.028 | 0.739-1.430 | 0.869 |
| *MCM2* | 0.959 | 0.615-1.497 | 0.855 |
| *NRTN* | 0.622 | 0.387-0.999 | 0.050 |
| *BARX2* | 0.953 | 0.783-1.158 | 0.626 |
| *CEACAM5* | 1.029 | 0.900-1.175 | 0.679 |
| *ORC6* | 1.346 | 0.803-2.258 | 0.259 |
| *TMPRSS2* | 0.983 | 0.770-1.255 | 0.890 |
| *SPAG5* | 0.875 | 0.530-1.443 | 0.600 |
| *TSPAN6* | 1.230 | 0.835-1.813 | 0.294 |
| *MCM4* | 0.785 | 0.498-1.236 | 0.295 |
| *PRC1* | 1.169 | 0.715-1.912 | 0.534 |
| *BIRC5* | 0.940 | 0.566-1.560 | 0.810 |
| *VSIG2* | 0.957 | 0.740-1.236 | 0.735 |
| *CDCA3* | 0.878 | 0.517-1.491 | 0.630 |
| *AURKB* | 0.709 | 0.480-1.045 | 0.082 |
| *TEDC2* | 0.539 | 0.321-0.905 | 0.019 |
| *CKS1B* | 0.822 | 0.483-1.399 | 0.470 |
| *KNSTRN* | 1.191 | 0.634-2.237 | 0.587 |

HR, hazard ratio; CI, confidence interval; Grey, *p* < 0.1.
